# Postpartum Opioid Use in the United States and the Implications to Maternal and Public Health: A Scoping Review

**DOI:** 10.1007/s10995-025-04163-x

**Published:** 2025-09-15

**Authors:** Allison N. Miller, Dennis E. N. Daniels, Sarah Cercone Heavey

**Affiliations:** 1 Department of Epidemiology and Environmental Health, School of Public Health and Health Professions, State University of New York at Buffalo, Buffalo, NY 14214 USA; 2 Department of Community Health & Health Behavior, School of Public Health & Health Professions, State University of New York at Buffalo, Buffalo, NY 14214 USA

**Keywords:** Postpartum opioid use, Postpartum opioid prescribing, Postpartum pain management, Maternal health, Public health

## Abstract

**Introduction/Purpose:**

Postpartum opioid prescription rates remain high, leading to increased morbidity and mortality and increased licit opioid medications diverted into communities. This scoping analysis examined the current processes of postpartum opioid prescribing patterns in America and the implications to maternal and public health.

**Methods:**

From the databases PubMed, Medline, and Web of Science, a scoping review was performed utilizing the PRISMA-ScR checklist (Tricco et al. in Ann Intern Med 169(7):467–473, 2018, 10.7326/M18-0850). The primary objective of the search strategy was to identify studies that focused on the postpartum timeframe (obstetric delivery to one year postpartum) and prescribed opioids.

**Results:**

A total of 26 articles met inclusion criteria. Articles were broken down into four themes: trends or current state of postpartum opioid prescribing practices (*n* = 7); postpartum opioid related risk factors (*n* = 6); rates of new persistent opioid use and opioid use disorder (OUD; *n* = 5); protocols or research into reducing postpartum opioid use (*n* = 8).

**Discussion/Conclusion:**

A variety of interventions and protocols have been found to be advantageous in reducing postpartum opioid use. Despite many of these successful efforts, postpartum opioid prescription rates remain high. Implementation of any number of interventions and protocols may be beneficial to reducing postpartum opioid use. Initiating a postpartum pain task force protocol (PPTFP) before obstetric delivery is recommended.

## Significance

Postpartum is characterized by substantial opioid use. Despite successful efforts to reduce postpartum opioid use, rates remain high, increasing likelihood of breakthrough pain, new persistent opioid use & OUD, harms related to opioid use, and opioid diversion. Escalation contributes to higher levels of prescribed opioids used nonmedically in communities.

To our knowledge, no study has utilized PPTFP and examined effects on opioid prescribing, and use, postpartum. This review found utilization of multiple interventions, including PPTFP, may significantly reduce postpartum opioid use while keeping patient pain expectation levels appropriate using functionality scales (e.g., ability to lift baby, ease of gentle movement).

## Introduction

Opioids are among the most misused prescription drugs with the potential to cause serious risks to patients and communities, including OUD and overdose deaths (Dowell et al., 2022). Obstetric delivery is the most frequent cause of hospital admission and cesarean section is the most common major surgery in the United States, making the postpartum period a time of high opioid prescribing and use (Dowell et al., 2022). Postpartum women are often prescribed opioids regardless of objective and subjective measures of pain, frequently more than what is necessary for pain control (Badreldin et al., 2018a). This overprescribing can lead to overconsumption by the patient, excess opioids being stored and potentially misused later, or diversion to the community (Bateman et al., 2017; Cohen et al., 2022).

This scoping review examined the current processes of postpartum opioid prescribing patterns in the United States and the implications for maternal and public health. The aim of this review is to identify trends, assess current regulations and policies, examine potential inequities and biases, evaluate the effects of postpartum opioid use, and provide recommendations to better ensure responsive practices with opioid prescriptions for postpartum patients.

## Methods

A review of the literature was conducted to explore the breadth of evidence and identify gaps in the current research landscape. This comprehensive review focused on a national level, aiming to synthesize a wide range of literature and evidence.

The primary objective was to identify studies on postpartum opioid prescribing and use from obstetric delivery to one year postpartum. To methodologically ground this review, the PRISMA-ScR checklist (Tricco et al., 2018) and a modified PRISMA-ScR flow diagram were utilized (see Fig. 2; Page et al., 2021). The search was developed utilizing University at Buffalo’s library of research and search tools as well as instruction from Sarah C. Heavey, PhD, Dennis E.N. Daniels, PhD, MPH, and Michelle Zafron, MLS. Themes and subthemes were created using a reflexive thematic analysis approach. All research, article screening, analysis, coding and categorization enacted by main author with guidance from additional authors.

### Eligibility Criteria

For inclusion, articles had to be from research conducted in the U.S., written in English, and comprising of data up to one-year postpartum with the earliest publication year of 2017 to focus on current research and balancing contemporary information with the evolving nature of opioid prescribing and use. Additionally, no articles were included before the 2017 publication date due to the evolving nature of the U.S. opioid epidemic and the publication of the CDC chronic pain prescribing guidelines in 2016 (Dowell et al., 2016).

Articles were excluded if the study exclusively reviewed antepartum and intrapartum periods, focused on substance use unrelated to opioids, included patients with pre-existing diagnoses of substance use disorder (SUD) or OUD, or focused on chronic pain patients or patients with extreme laceration/tearing during childbirth.

Eligible studies included retrospective cohort studies, prospective cohort analyses, quantitative research, quasi-experimental studies, government reports, prospective quality improvement studies, clinical consensus research, and clinical trials. Articles focused on postpartum opioid prescribing trends, risk factors, rates of new persistent opioid use and OUD, and protocols to reduce postpartum opioid use.

### Selection of Sources of Evidence

**Fig. 1:**
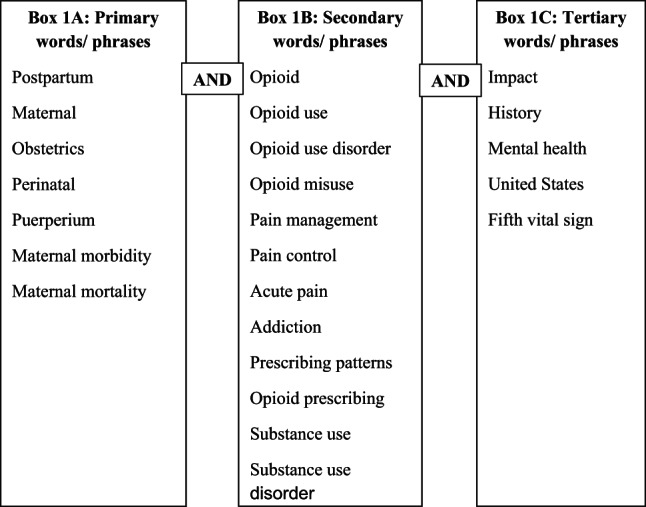
Search words and phrases used in this scoping review. The boxes represent the frequency of the search terms usage from primary (most used), to secondary (less used), to tertiary (least used). AND was used between all search words/phrases

**Fig. 2:**
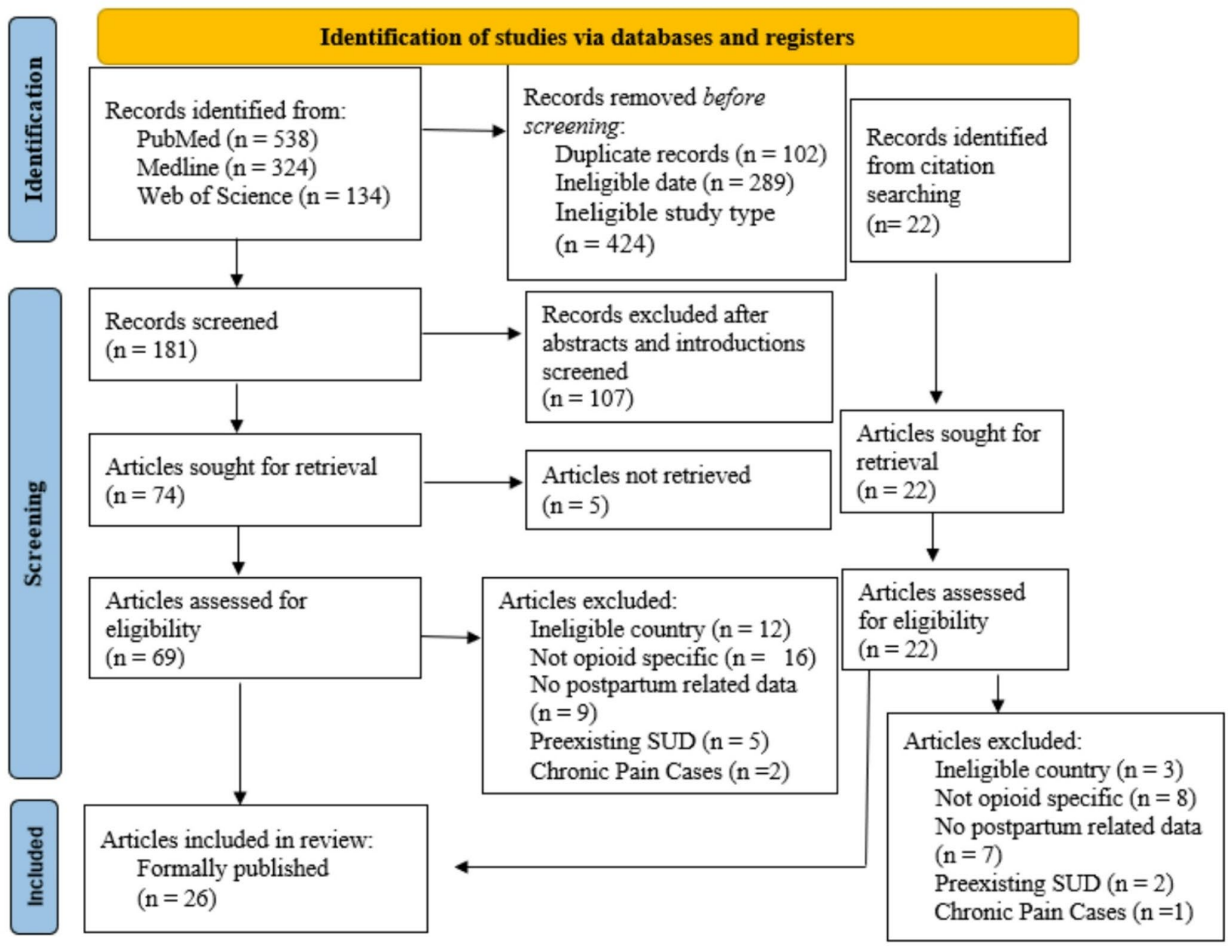
PRISMA Sc-R (modified) flow diagram. This figure demonstrates the PRISMA flow for articles included in this review, as well as the number of abstracts or articles screened/reviewed at each step. *From*: Page et al., (2021)

A visual depiction of the source selection process can be found in Figs. 1 and 2. Utilizing PubMed, Medline, and Web of Science, search terms and filters limited sources to an earliest publication date of 2017. Sources were eliminated immediately if the title depicted pregnancy data not related to postpartum or included the phrases “systematic review” or “scoping review”. Titles, abstracts, and introductions were screened for relevance.

Articles were categorized into four major themes using reflexive thematic analysis with inductive coding and categorization based from examining the focus of each study. However, some articles touched on multiple major themes, and most articles discussed multiple similar subthemes. Thus, articles were categorized into eight additional subthemes to further deduce evidence.

## Results

The review included seventeen retrospective cohort studies, two prospective cohort analyses, one quantitative study, one quasi-experimental study, two government reports, one prospective quality improvement study, one clinical consensus research study, and one clinical trial. These studies reported results from 2,432,146 patients, with sample sizes ranging from 50 to 768,455. Six studies included vaginal births, nine included cesarean births, and eleven included both.

### Trends of Postpartum Opioid Prescribing Patterns (Table 1)

**Table 1:** Trends evidence table

Citation	Research method	Data collection	Sample size	Aims/purpose	Scope and significance
Badreldi et al. (2022)	Retrospective cohort study	pre- ACOG, CDC (2022) pre & post- CDC (2016)	10,242 patients	To evaluate whether patient-prescriber racial and ethnic concordance is associated with postpartum opioid prescribing patterns and patient-reported pain scores	Data have consistently shown that patients of racial and ethnic minority groups receive less opioid pain treatment compared to non-Hispanic white (NHW) patients. Notably, recent data have shown that racial and ethnic disparities in postpartum opioid prescribing exist
Badreldin et al. (2018a)	Retrospective cohort study	pre- ACOG, CDC (2022) pre & post- CDC (2016)	12,326 patients	We sought to describe opioid prescribing patterns at the time of discharge following delivery in a large, diverse cohort, and to describe the relationship of these patterns with objective and subjective measures of pain prior to discharge	The amount of prescription opioids did not vary by objective or subjective reports of pain. Postpartum women are commonly prescribed opioids at hospital discharge, but the quantity does not appear to be based on subjective or objective measures of pain at the time of discharge
Badreldin et al. (2018b)	Retrospective cohort study	pre- ACOG, CDC (2022) pre & post- CDC (2016)	9,038 patients	Our objectives were as follows: (1) to describe the use of opioids during the last 24 hours of postpartum hospitalization in women following vaginal delivery and (2) to identify patient and provider characteristics associated with the use of opioids during this time period.	Following a vaginal delivery, 24.8% of women utilized an opioid during the last 24 hours of postpartum hospitalization. Greater use of acetaminophen and having had orders written by an advanced practitioner were associated with a decreased likelihood of using an opioid
Dowell et al. (2022)	Government report	N/A	N/A	This clinical practice guideline expands and updates the 2016 CDC Opioid Prescribing Guideline to provide evidence-based recommendations for prescribing opioid pain medication for acute, subacute, and chronic pain for outpatients aged ≥ 18 years	This update leverages new data to expand content on prescription opioids for acute and subacute pain throughout the recommendations. Importantly, the update also aims to clearly delineate recommendations that apply to patients who are being considered for initial treatment with prescription opioids and patients who have been receiving opioids as part of their ongoing pain management
Jarlenski et al. (2017)	Retrospective cohort study	pre- CDC (2016), ACOG	164,720 women	To estimate the prevalence of filled opioid prescriptions after vaginal delivery	An average of 39% of Medicaid-enrolled women of reproductive age fill an outpatient prescription for opioid pain relievers each year. In the last decade, overdose deaths from opioid pain relievers have increased fivefold among U.S. women
McKinnish et al. (2021)	Retrospective cohort study	pre- CDC (2022) post- CDC (2016), ACOG	416 patients	This study aims to investigate the effect of race on inpatient postpartum opioid consumption and the amount of opioids prescribed at discharge after vaginal or cesarean delivery	Numerous studies have shown significant racial and ethnic disparities in opioid prescribing practices. Furthermore, though Black and Hispanic women report higher pain scores in the postpartum period, they receive fewer inpatient morphine milligram equivalents (MMEs) compared to white women
Ravikanti et al. (2022)	Retrospective cohort study	pre- CDC (2022) post- CDC (2016) pre & post- ACOG	411 patients	This study aimed to determine if there were differences in prescribing practices based on the specific post-operative day that opioid prescriptions were written, and if there were differences in the prescribing practices for cesarean deliveries following the publication of American College of Obstetrics and Gynecologists (ACOG) Committee Opinion 742	Patients whose deliveries occurred after the publication of ACOG Committee Opinion 742 received discharge prescriptions with fewer MMEs, fewer doses per day, and the same dosage than those whose deliveries occurred before the publication, reflecting the overall national trend of decreasing prescription opioids over these years

Studies on postpartum opioid prescribing trends varied in focus, including inpatient or outpatient settings, type of delivery, location, race and published prescribing guidelines.

#### Trends Varying on Patient Setting and Delivery Type

For inpatient opioid prescribing post vaginal delivery, 24.8% of patients used opioids in the last 24 h of hospital stay (Badreldin et al., 2018b). Inpatient opioid use post vaginal delivery was common, even in uncomplicated cases. Factors linked to reduced inpatient opioid use were Asian race/ethnicity, marital status, increased acetaminophen and NSAID use, and orders from advanced practitioners (typically, midwives).

Outpatient opioid prescribing post vaginal delivery occurred at lower rates than inpatient, but prescriptions were available for a longer period. For example, 12% of patients filled an opioid prescription within five days of delivery, with 14% of those patients filling a second prescription six to 60 days later (Jarlenski et al., 2017).

Opioids prescribed for hospital discharge were three times more common for caesarean birth compared to vaginal. A study found that 30% and 87% of patients, respectively, were prescribed opioids at hospital discharge following vaginal and cesarean sections (Badreldin et al., 2018a). The quantity of opioids prescribed was similar following either vaginal or cesarean delivery regardless of objective and subjective pain measures prior to discharge.

#### Geographic Variation

Geographic variations in postpartum opioid prescribing were significant across states, with notable differences in rates and durations. For instance, among women with uncomplicated vaginal deliveries, there was a 7-fold difference in prescribing rates (7.6 to 53.4%), a 5-fold difference in prescriptions lasting more than five days (5.1 to 25.7%), and a 19% absolute difference in prescriptions exceeding 280 morphine milligram equivalents (MME) across states (Becker et al., 2018). To note, this study is one that could have fit into multiple themes due to the significant variation in evidence; additional information located under the *risk factors* theme.

#### Racial and Ethnic Variance

Studies showed data variation on trends in opioid prescribing patterns related to race. Badreldin et al. (2022) concluded that opioid prescribing and pain perception were not ameliorated by patient-prescriber racial and ethnic concordance, such that when patients and prescribers were the same race/ethnicity, opioid prescribing and pain perception still did not match (e.g., non-Hispanic Black provider and patient had X discordance). Further, non-Hispanic Black and Hispanic patients had 27% decreased odds of receiving an opioid prescription at postpartum discharge compared to non-Hispanic white patients, despite reporting higher pain scores. An outlier study, McKinnish et al. (2021), concluded opioid tablets prescribed at discharge had no association with patient race.

#### Prescribing Guidelines

Outpatient opioid prescribing post cesarean section was examined pre- and post-publication of The American College of Obstetrics and Gynecologists (ACOG) Committee Opinion 742 (ACOG, 2018). This publication provided framework for management of acute perineal, uterine, and incisional pain. After the publication there was a decrease from 93.9 to 63.9% in opioid prescriptions at discharge (Ravikanti et al., 2022).

More broadly, the CDC released guidelines for prescribing opioids for pain in 2022 (Dowell et al., 2022). This publication highlighted a decrease in reported misuse of prescription drugs from 12.5 million cases in 2015 to 9.7 million cases in 2019. These guidelines stress individualized patient care, enhanced communication between clinicians and patients for shared decision-making, and risk reduction in opioid pain therapy (Dowell et al., 2022). Notably, these guidelines did not specifically address obstetric care but were mentioned here as the only recent government opioid prescribing guidelines.

### Postpartum Opioid Related Risk Factors for Maternal and Public Health (Table 2)

**Table 2:** Risk evidence table

Citation	Research method	Data collection	Sample size	Aims/purpose	Scope and significance
Becker et al. (2018)	Retrospective cohort study	pre- CDC (2016), ACOG	871,195 deliveries among 768,455 women	Describe state- and census division-level variation in opioid analgesic use after discharge following uncomplicated vaginal delivery in the United States. Describe census division- and state-level variation in opioid prescription duration and dose as well as to illustrate national level temporal trends in opioid prescription rate and dose following vaginal delivery	Our analysis is the first to demonstrate the national variation in opioid prescribing patterns after obstetric delivery. We show that nearly 30% of women fill an opioid prescription following hospital discharge after an uncomplicated vaginal delivery, and state-level rates of opioid prescribing differ by seven-fold among this group. Physicians routinely prescribe far more opioids for acute post-surgical pain than patients actually use
Horn et al. (2022)	Retrospective cohort study	pre- CDC (2016), ACOG	264,135 patients	To examine the association between postpartum opioid prescription fills and the one-year risk of all-cause mortality among women with live births	Pregnancy-associated deaths, or deaths from any cause while pregnant or within the first year after delivery, have risen in recent decades. Filling two or more opioid prescriptions during the postpartum period was associated with a significant increase in one-year risk of death among new mothers
Nidey et al. (2020)	Retrospective cohort study	pre- ACOG, CDC (2022) pre & post- CDC (2016)	25,279 women	The objective of this study was to examine the association of mood and anxiety disorders with filling opioid prescriptions within the first 3 months postpartum	Mental health emerged as an important risk factor for opioid use, especially among women who delivered vaginally. This may be attributed to prescribing patterns based on delivery type, as women with cesarean deliveries are routinely prescribed at least one opioid prescription which reduces the effect of mental health conditions on opioid fills
Stewart et al. (2023)	Government report	pre- CDC (2022) post- CDC (2016), ACOG	1,990 patients	The use and evaluation of the Pregnancy Risk Assessment Monitoring System (PRAMS) to address the association of mental health conditions, recent stressful life events, and adverse childhood experiences with postpartum substance use	Most pregnancy-related deaths due to mental health conditions, which include overdose and poisoning related to substance use disorder, occur during the late (43–365-day) postpartum period. Adverse childhood experiences and stressful life events are associated with increased substance use during pregnancy
Wiese et al. (2021a)	Retrospective cohort study	pre- CDC (2016), ACOG	65,170 women	We examined the association between the dosage of the first opioid prescription filled after cesarean delivery and the risk of serious opioid-related events	Opioid-naïve women who filled a postpartum opioid prescription at commonly prescribed doses after cesarean delivery had an increased risk of serious opioid-related events compared to women who did not fill a postpartum opioid prescription. Low opioid doses were not associated with a significant increase in the risk of serious opioid-related events
Wiese et al. (2021b)	Retrospective cohort study	pre- CDC (2016), ACOG	113,834 women	We sought to test the hypothesis that among pregnant women who had a vaginal delivery, the risk of serious opioid-related events differed by the dose of their first postpartum opioid prescription	The prescribing rates after vaginal delivery vary markedly by country, region, and hospital, suggesting that postpartum opioid prescribing decisions are influenced by factors other than patient need, including provider preference and nonclinical patient factors (maternal age, race/ethnicity, insurance provider, rurality of residence, and distance to hospital)

Studies discussing postpartum opioid related risk factors (serious opioid-related events, opioid misuse, increased opioid use) varied in topic from delivery type, opioid dosage, individual factors, and region of residence.

#### Delivery Type

Results showed that 53.2% of patients following vaginal deliveries filled opioid prescriptions within four days, with an increased risk of serious opioid-related events at 2.40 per 100 person-years regardless of dose (Wiese et al., 2021a). For cesarean deliveries, 87.0% filled an outpatient opioid prescription within five days after delivery, with an overall rate of serious opioid-related events was 3.0 per 100 person-years (Wiese et al., 2021b).

#### Opioid Dosage

The secondary analysis of the Wiese et al. (2021b) study showed that with a lower opioid dosage (5 mg oxycodone tablets and less than 10 tablets in total) there was not an increased risk of serious opioid events. Examining postpartum opioid prescription fills and mortality risk showed patients with two or more prescriptions had a higher mortality rate of 120.5 per 100,000 person-years compared to those with one or fewer refills at 57.7 per 100,000 person-years (Horn et al., 2022). Overall, patients who filled two or more postpartum opioid prescriptions had a 46% increased risk of death.

#### Individual Factors

Patients at higher risk for potential opioid misuse were identified as people who had previously used tobacco, opioids, antidepressants, or benzodiazepines, had > 1 outpatient visits during pregnancy (including, but not limited to prenatal care visits), and were white (Wiese et al., 2021b). Additionally, patients at increased risk for opioid use also commonly used ADHD medications and antipsychotics, and were more likely to be diagnosed with depression, anxiety, and injuries from accidents and trauma (Horn et al., 2022). Patients with mood and anxiety disorders were at a higher risk of filling opioid prescriptions post-delivery, with a 50% increase for vaginal deliveries and 20% for cesarean deliveries (Nidey et al., 2020). Patients with anxiety or depression were nearly twice as likely to fill two opioid prescriptions, while those with bipolar disorder had a 3.80 times greater risk.

Continuing, a government report published by Stewart et al. (2023) using the Pregnancy Risk Assessment Monitoring System (PRAMS) in seven high opioid overdose mortality states aimed to understand opioid use risks among postpartum patients with similar results (twice the prevalence of substance use in respondents with depressive symptoms, current depression, or anxiety). Moreover, experiencing six or more stressful life events in the year before giving birth or having four adverse childhood experiences related to household dysfunction significantly increased postpartum substance use.

#### Region of Residence

Postpartum patients living in the East South-Central region of the United States are prescribed the highest in opioid rates and duration and experienced the highest risk of opioid-related events postpartum with an overall usage rate of 46.8% (Becker et al., 2018). Alabama and Mississippi have the highest rates of opioid prescriptions following uncomplicated cesarean sections (49.9% and 47.2%, respectively), as well as higher rates of prescriptions lasting longer than five days (15.4% and 16.2%; Becker et al., 2018).

### Rates of New Persistent Opioid Use and OUD (Maternal and Public Health) (Table 3)

**Table 3:** New persistent opioid use and OUD

Citation	Research method	Data collection	Sample size	Aims/purpose	Scope and significance
Bateman et al. (2017)	Quantitative research	pre- CDC (2016), ACOG	720 patients	To define the amount of opioid analgesics prescribed and consumed after discharge after cesarean delivery	The amount of opioid prescribed after cesarean delivery generally exceeds the amount consumed by a significant margin, leading to substantial amounts of leftover opioid medication. Lower opioid prescription correlates with lower consumption without a concomitant increase in pain scores or satisfaction
Cohen et al. (2022)	Prospective cohort analysis	pre- CDC (2022) post- CDC (2016), ACOG	205 patients	Our primary aim was to assess post‐cesarean outpatient opioid use. A secondary aim was to identify characteristics associated with opioid use. We hypothesized that patients would consume fewer opioids than prescribed	In our study, providers overprescribed opioids in the excess of five times that reportedly consumed by patients. This is consistent with results of other studies and supports the need to change prescribing patterns
Peahl et al. (2019)	Retrospective cohort study	pre- ACOG, CDC (2022) pre & post- CDC (2016)	308,226 patients	To assess the association between opioid prescribing administered for vaginal or cesarean delivery and rates of new persistent opioid use among women	Based on the present findings, roughly 77,000 of the 3.86 million women who deliver each year are at risk of continued opioid use. Our findings highlight that a substantial number of patients continue to fill opioid prescriptions long after expected recovery
Peahl et al. (2020)	Retrospective cohort study	pre- ACOG, CDC (2022) pre & post- CDC (2016)	158,425 patients	To evaluate the association between opioid prescribing during pregnancy and new persistent opioid use in the year following delivery	As maternity care providers have recognized the role of peripartum opioid prescribing in the opioid epidemic, reducing opioid prescribing following delivery has become a national priority. This increased focus is warranted, as 1 in 75 women in the United States who fill an opioid prescription in the peripartum period will continue filling prescriptions up to 1 year postpartum. In fact, exposure to postpartum opioids has been linked to new persistent use after delivery, independent of the type of birth (vaginal vs cesarean delivery), suggesting that the risk is inherent to the opioid prescription
Zhu et al. (2021)	Retrospective cohort study	pre- CDC (2016), ACOG	459,829 patients	To evaluate the effect of receiving an opioid prescription after vaginal delivery on the risk of subsequent persistent opioid use, opioid use disorders and overdose	Receiving an opioid prescription after vaginal delivery was associated with a 2.5-fold increased risk for persistent opioid use, and a near 2-fold increased risks for opioid use disorder and opioid overdose

Studies discussing rates of new persistent opioid use and OUD varied on subject matter related to vaginal delivery, cesarean delivery and overprescribing, and implications to opioid prescribing and filling timing.

#### Vaginal Delivery Rates

Focusing on persistent opioid use post-vaginal delivery (defined as 10 opioid fills or > 120 days supply dispensed from 30 to 365 days after delivery), according Zhu et al. (2021), 30.6% of patients receive opioids after delivery with findings showing a 2.5-fold increased risk for persistent opioid use, and a near 2-fold increased risks for opioid use disorder and opioid overdose. Estimating out of approximately 2.5 million vaginal deliveries in the US in 2018, with around 750,000 opioid prescriptions dispensed, there could be an additional 16,575 cases of persistent opioid use, 6,075 cases of opioid use disorder, and 150 cases of opioid overdose attributed to postpartum opioid use (Zhu et al., 2021).

#### Cesarean Delivery Overprescribing

Two articles targeting cesarean deliveries addressed overprescribing opioids postpartum, leading to increased risks of diversion and nonprescribed opioid consumption, thus increasing persistent opioid use and opioid use disorder rates. One revealed 73% of patients filled a postpartum opioid prescription, but 42% did not consume the opioids prescribed, with providers overprescribing opioids in the excess of five times that reportedly consumed by patients (Cohen et al., 2022). The second found that 85.4% of patients filled an opioid prescription post-cesarean delivery, with a median of 40 tablets dispensed, 20 consumed, and 16 leftover (Bateman et al., 2017). Most patients (95.3%) did not dispose of their excess medications according to patient reports. Regardless of pain levels or patient satisfaction, higher prescribed amounts led to increased opioid consumption, shaping patient expectations on usage. These studies highlighted that approximately 20 million opioid tablets enter communities annually from leftover medications post-cesarean deliveries, significantly contributing to the theoretical availability of opioids for diversion or misuse (Bateman et al., 2017).

#### Implications to Opioid Prescribing and Filling Timing

Examining the association between filling an opioid prescription before delivery and new persistent opioid use postpartum (defined by both Peahl et al. articles as pharmacy claims for one or more opioid prescription four to 90 days after discharge and one or more prescription 91 to 365 days after discharge), 27.0% of patients with vaginal deliveries and 75.7% with cesarean deliveries filled postpartum opioid prescriptions, with 1.7% and 2.2% respectively developing new persistent opioid use (Peahl et al., 2019). Peahl et al. (2019) included additional factors linked to persistent opioid use not mentioned prior including receiving a pre-delivery prescription and prescriptions equal to or exceeding 225 MME. Between 14% and 22% of patients filled an opioid prescription during pregnancy, with 4% developing new persistent opioid use postpartum (Peahl et al., 2020). Combining the data, an estimated 77,000 out of the 3.86 million women who deliver annually are at risk of continued opioid use.

### Protocols and Research in Postpartum Opioid Use Reduction (Table 4)

**Table 4:** Interventions/protocols evidence table

Citation	Research method	Data collection	Sample size	Aims/purpose	Scope and significance
Bornstein et al. (2020)	Retrospective cohort study	pre- CDC (2022) post- CDC (2016) pre & post- ACOG	12,898 cesarian births	The purpose of our study was to evaluate the potential impact of adopting a standardized order set based on multimodal combination analgesic therapy on both opioid usage as well as quality of pain control in the immediate post cesarean delivery period in a large healthcare system with multiple obstetrical units	Multimodal analgesic therapy for post-cesarean pain management reduces inpatient opioid use while improving pain control. Incorporation of a multimodal order set as a default in the EMR facilitates effective and widespread implementation on a large scale
Bryant and Miller (2021)	Clinical consensus	pre- CDC (2022) pre & post- CDC (2016), ACOG	77 records	To recommend a pharmacologic stepwise multimodal approach for postpartum pain management	Pain can interfere with individuals’ ability to care for themselves and their infants, and untreated pain is associated with risk of greater opioid use, postpartum depression, and development of persistent pain. Clinicians should therefore be skilled in individualized management of postpartum pain
Holland et al. (2019)	Quasi-experimental study	pre- ACOG, CDC (2022) post- CDC (2016)	372 patients	To evaluate the effects of eliminating the routine use of oral opioids for post cesarean delivery analgesia on post cesarean opioid consumption	Eliminating routine ordering of oral opioids after cesarean delivery is associated with a significant decrease in opioid consumption while maintaining the same levels of pain control and patient satisfaction. Oral opioids are not needed by a large proportion of women after cesarean delivery
Macias et al. (2022)	Prospective cohort study	pre- CDC (2022) post- CDC (2016), ACOG	378 women	To determine if a multimodal pain management regimen after cesarean delivery reduces the required number of morphine milligram equivalents (a unit of measurement for opioids) compared with traditional morphine patient-controlled analgesia while adequately controlling postoperative pain	Transition to a multimodal pain management regimen for women undergoing cesarean delivery resulted in a decrease in opioid use while adequately controlling postoperative pain. A multimodal regimen was associated with early successful exclusive breastfeeding
Nakahara et al. (2018)	Retrospective cohort study	pre- ACOG, CDC (2022) post- CDC (2016)	187 patients	Our study aimed to determine the routine prescribing practices of providers at our institution for patients after vaginal deliveries and the number of encounters for pain these patients initiated during the postpartum period	Our data suggest that despite the likely excessive amount of narcotics given on discharge after uncomplicated vaginal deliveries, approximately 1 in 5 patients initiates an encounter to address postpartum pain prior to her regularly scheduled postpartum visit. In addition, parity appears to play a role in postpartum narcotic usage in the hospital, possibly because of patients’ prior experiences with the use of narcotics for pain control in postpartum care
Prabhu et al. (2017)	Clinical trial	pre- ACOG, CDC (2022) post- CDC (2016)	50 patients	To assess whether a shared decision making intervention decreases the quantity of oxycodone tablets prescribed after cesarean delivery	A shared decision-making approach to opioid prescribing after cesarean delivery was associated with approximately a 50% decrease in the number of opioids prescribed postoperatively in this cohort compared with our institutional standard prescription
Prabhu et al. (2018)	Prospective quality improvement study	pre- ACOG, CDC (2022) post- CDC (2016)	624 cesarean deliveries	To assess whether a multiphase, departmental quality improvement effort decreases opioid prescribing and increases multimodal analgesic use after cesarean delivery	In a nationwide survey of women undergoing cesarean delivery, 85% received an opioid prescription upon discharge but only consumed half of the median discharge prescription of 40 tablets. Moreover, a higher number of opioid tablets prescribed was not associated with improved pain control, higher patient satisfaction, or lower refill rates, but was associated with increased consumption
Rogers et al. (2019)	Retrospective cohort study	pre- ACOG, CDC (2022) post- CDC (2016)	14,419 patients	To estimate the effects of an inpatient initiative to decrease opioid use among women admitted to labor and delivery	In an observational cohort study of 246 women, women with high inpatient opioid use were 60% more likely to be high users in the outpatient setting. Postpartum outpatient opioid prescribing has been found to be common, and discharge prescribing practices are unstandardized and amenable to reduction through shared decision making

Studies focusing on protocols and research in postpartum opioid use reduction varied on topics ranging from patient demand for opioids, quality improvement initiatives, and multimodal pain management approaches.

#### Patient Demand

Focusing on patient demand for improved pain control postpartum, Nakahara et al. (2018) analyzed 187 patients, receiving a total of 3,635 narcotic pills at discharge (5/ 325-mg oxycodone/acetaminophen), averaging 19.4 pills per patient. Despite this prescription amount, patients frequently sought additional pain management, resulting in approximately one encounter per five patients contacting physicians through calls, emails, or emergency room visits.

#### Quality Improvement Initiatives

In a series of quality improvement initiatives, researchers educated patients on pain management strategies following cesarean deliveries. Patients were empowered to choose their prescribed opioid quantities, leading to a 50% reduction in postoperative opioid prescriptions and high patient satisfaction rates (Prabhu et al., 2017). Another initiative involved patient education and shared decision-making to optimize opioid use, resulted in a decrease from 33.2 to 21.5 opioid tablets per patient after two phases (Prabhu et al., 2018). Ibuprofen and acetaminophen usage increased significantly during these phases. A third initiative focused on reducing routine opioid prescriptions post-cesarean delivery, resulting in decreased in-hospital opioid usage from 68 to 45% and discharge prescriptions from 91 to 40% (Holland et al., 2019). Patient satisfaction with pain relief remained consistent throughout these changes.

#### Multimodal Pain Management

Multimodal pain management approaches for post-delivery pain control were analyzed in numerous studies, including a clinical consensus published by ACOG (Bryant & Miller, 2021). This publication emphasized the importance of using nonopioid analgesics as first-line pain management for both vaginal and cesarian sections, borrowing from the stepwise analgesic approach for cancer pain published by the World Health Organization. Further stated, when opioids are needed, low-dose, low-potency, and short-acting opioids should be used first, only to be followed by stronger opioids when patients are experiencing refractory or breakthrough pain in the postpartum era. An analysis on cesarean deliveries reported a significant reduction in MME from 128 to 28 within 48 h post-delivery after implementing a multimodal pain management protocol, with improved pain scores (Macias et al., 2022). Post-cesarean pain management showed decreased opioid use, reduced opioid doses, increased acetaminophen use, and reduced severe pain scores (Bornstein et al., 2020). Additional evaluations of both vaginal and cesarean deliveries found reductions in opioid use and MME, but noted lower patient-reported pain control satisfaction (Rogers et al., 2019). While the Rogers et al. (2019) study educated healthcare providers on multimodal pain management, it lacked patient education on opioid risks and functional goal assessment for pain management.

### Subthemes (Tables 5, 6, 7 and 8)

**Table 5:** Subthemes of trends articles

	Subthemes	Variations in opioid prescribing rates	Racial and ethnic variations in prescribing practices	Opioid prescribing in excess	Mental health analysis	Opioid tablet strength and quantity	Shared decision making	Postpartum opioid prescribing standardization	Multimodal pain management regimens
Trends									
Badreldin et al. (2022)		✓	✓					✔	
Badreldin et al. (2018a)		✔		✔		✔		✔	
Badreldin et al. (2018b)		✔							✔
Dowell, D. et al. (2022)							✔		✔
Jarlenski et al. (2017)		✔	✔					✔	
McKinnish et al. (2021)			✔						
Ravikanti et al. (2022)		✔				✔			

**Table 6:** Subthemes of risk articles

	Subthemes	Variations in opioid prescribing rates	Racial and ethnic variations in prescribing practices	Opioid prescribing in excess	Mental health analysis	Opioid tablet strength and quantity	Shared decision making	Postpartum opioid prescribing standardization	Multimodal pain management regimens
Risk									
Becker et al. (2018)		✔		✔		✔		✔	
Horn et al. (2022)		✔			✔	✔	✔		
Nidey et al. (2020)					✔				
Stewart et al. (2023)		✔	✔		✔				
Wiese et al. (2021a)		✔			✔	✔			
Wiese et al. (2021b). “Prescription..”			✔		✔	✔	✔		

**Table 7:** Subthemes of new persistent opioid use and OUD articles

	Subthemes	Variations in opioid prescribing rates	Racial and ethnic variations in prescribing practices	Opioid prescribing in excess	Mental health analysis	Opioid tablet strength and quantity	Shared decision making	Postpartum opioid prescribing standardization	Multimodal pain management regimens
New persistent opioid use & OUD									
Bateman et al. (2017)				✔					
Cohen et al. (2022)				✔				✔	
Peahl et al. (2019)		✔		✔	✔	✔	✔	✔	✔
Peahl et al. (2020)					✔				
Zhu et al. (2021)		✔	✔	✔	✔				

**Table 8:** Subthemes of interventions/protocols articles

	Subthemes	Variations in opioid prescribing rates	Racial and ethnic variations in prescribing practices	Opioid prescribing in excess	Mental health analysis	Opioid tablet strength and quantity	Shared decision making	Postpartum opioid prescribing standardization	Multimodal pain management regimens
Interventions/protocols									
Bornstein et al. (2020)								✔	✔
Bryant and Miller (2021)			✔				✔		✔
Holland et al. (2019)				✔					
Macias et al. (2022)									✔
Nakahara et al. (2018)				✔					
Prabhu et al. (2017)							✔		
Prabhu et al. (2018)						✔	✔		✔
Rogers et al. (2019)							✔	✔	✔

The main four themes stemming from the reflexive thematic analysis described the key data results from the literature, however, since many studies and publications touched on multiple subjects, the following eight subcategories were configured to further analyze the data: variations in opioid prescription rates (12 studies), racial and ethnic variations in prescribing practices (7 studies), opioid prescribing in excess of patient consumption (8 studies), mental health considerations (9 studies), opioid tablet strength and quality (8 studies), shared decision making (8 studies), postpartum opioid prescribing standardization (8 studies), multimodal pain management regimens (8 studies). Visual representations of all studies are broken down into themes and subthemes shown in Tables 5, 6, 7 and 8. Due to the high volume of information and importance, each of these subthemes could be further examined with separate publications of the results. The point of categorization of these subthemes was to highlight the additional key topics being researched and discussed within current literature.

## Discussion

This scoping review broadly analyzed current postpartum opioid prescribing in the United States. It showed that many changes, including the ACOG Committee Opinion 742 and the CDC Clinical Practice Guideline for Prescribing Opioids for Pain have positively impacted patient and community health (ACOG, 2018; Dowell et al., 2022). Both publications emphasized individualized care, enhanced patient-provider communication for shared decision-making, and multimodal pain plans. Continued research and changes are needed to further improve postpartum health and reduce leftover opioid medications. It is important to note that seven of the 26 articles reviewed collected data before the published 2016 CDC guidelines, seventeen articles collected data before the ACOG Opinion 742 publication, and all articles collected data before the published CDC guidelines in 2022. However, the overarching themes and subthemes found with the data were the same as the themes and subthemes addressed within those publications. Individual article data collection details regarding guideline publications can be found in Tables 1, 2, 3 and 4.

This review showed that postpartum opioid prescribing remains common, with many researchers noting high prescribing rates despite increased awareness of opioid harms through studies and guidelines. Additionally, many studies revealed that physicians often overprescribe opioids, with high percentages of prescribed opioids going unused. Research has proven that increased opioid tablet strength and quantity correlate with increased harms postpartum. It is important to note that opioid use remains crucial for reducing pain postpartum. Historically, female reproductive pain has been undertreated. Furthermore, racial and ethnic disparities in opioid prescribing reveal that pain for patients of color has been systematically undertreated.

This analysis found significant variability in prescribing practices across geographic, socioeconomic, mental health, and racial/ethnic factors (Badreldin et al., 2022; Jarlenski et al., 2017). Racial and ethnic health disparities were linked to poor communication at patient, provider, and system levels (Badreldin et al., 2022). Researchers suggested that disparities in postpartum pain management might be due to structural or systemic factors. To enhance healthcare provider continuity, compliance, and mitigate implicit biases, obstetric standardized protocols are recommended, such as multimodal pain plans focusing on non-opioid pharmacologic management (Badreldin et al., 2022; Bornstein et al., 2020; Cohen et al., 2022). These protocols support the ongoing use of non-opioid medications to reduce breakthrough pain that may necessitate opioid intervention and potentially diminish racial and ethnic disparities by minimizing individual provider variations (Badreldin et al., 2022; Bornstein et al., 2020). The evidence within this analysis showed that standardized protocols, such as multimodal pain plans, work best when coupled with patient education and shared decision making between patient and provider (ACOG, 2018; Ravikanti et al., 2022). This not only increases patient understanding and communication between patient and provider but decreases patient demand for increased opioid use due to increased patient satisfaction (Bryant & Miller, 2021; Nakahara et al., 2018). Additionally, providing comprehensive information on pain management options empowers informed decisions, enhancing patient-provider communication and autonomy. Equipping obstetric patients with the necessary tools has been shown to notably decrease opioid use without compromising patient satisfaction (Ravikanti et al., 2022). More research is needed to define appropriate postpartum opioid usage rates. Further, there remains a need for opioid harm reduction, especially for those patients with increased risk factors related to opioid harms.

The findings and objectives of this analysis do not suggest that all prescribing providers are overprescribing opioids or that the only solution is to prescribe less and reduce provider autonomy. Postpartum care and pain management are individualized experiences influenced by biological, psychological, and social factors, and cannot be managed under a single protocol, policy, or guideline (Dowell et al., 2022). The main goal of this analysis was to find ways to increase responsible opioid prescribing to lower postpartum opioid use and harms without reducing patient satisfaction or provider autonomy.

A crucial research gap identified is the lack of studies on the impact of an antepartum patient educational course on postpartum pain management and opioid use. Peahl et al. (2019) recommended opioid-sparing protocols with patient preparation to set pain expectations and develop coping techniques. Bateman et al. (2017) emphasized the importance of educating patients on disposing of leftover opioid pills. Horn et al. (2022) suggested counseling on opioid risks and non-narcotic options. Prabhu et al. (2018) highlighted the need for patient education and involvement in prescribing opioid quantities. Rogers et al. (2019) attributed their success in reducing opioid use to redefining recovery by functional milestones and providing standardized order sets for analgesic needs. They noted the absence of patient education on opioid risks and functional goals. Incorporating this education could lead to greater acceptance of mild pain. Therefore, it is recommended to implement an antepartum educational course in the third trimester, focusing on postpartum pain management, known as the postpartum pain task force protocol (PPTFP).

The protocol could be developed in diverse ways to be taught by varying healthcare providers (e.g. RN, MD, CNM) depending on costs, institutional requirements, or other external factors. This educational session would take place at the physician’s office during a regular appointment and cover comprehensive pain management options. Patients would receive detailed information on the short and long-term effects of each option, including implications for breastfeeding. Furthermore, patients would be educated in assessing pain levels using functional measures (e.g. ambulation, holding infant, urination/defecation), rather than a traditional 1–10 pain scale and need for proper left-over opioid disposal. Additionally, this protocol is a low threshold education program that providers could work on with patients with the potential to be a feasible, standalone program. Research suggests that informed and empowered patients tend to make decisions that minimize harm (Prabhu et al., 2017). Pre-delivery education on postpartum pain control allows patients to make autonomous, well-informed decisions and understand the natural pain experiences associated with labor and delivery, whether vaginal or cesarean, and to measure pain levels utilizing a functionality scale.

### Limitations

The literature on opioid use in America is extensive, particularly concerning postpartum opioid prescribing patterns. While this review focused specifically on postpartum opioid prescribing, the abundance of studies and articles in this area presents a challenge. Further, pain, being subjective and unique to everyone, adds complexity to the analysis. Moreover, most studies in this review are retrospective analyses rather than randomized controlled trials (RCTs), limiting their strength, however RCTs could be difficult and potentially unethical with this area. The advantage to this review is the variety of literature included synthesized a broad scope to the current research and evidence to identify future empirical work.

## Conclusion

Despite various interventions and guidelines aimed at reducing postpartum opioid prescribing, it remains a routine part of the postpartum experience, leading to concerns such as breakthrough pain, persistent opioid use, opioid use disorder (OUD), and harm related to opioid use, including increased opioid diversion within communities. The postpartum period is often a patient’s initial exposure to opioids and poses a significant risk for misuse due to hormonal changes, sleep deprivation, lack of support, heightened pain levels, and lifestyle adjustments. Continued research and initiatives are crucial to reducing postpartum opioid use while maintaining patient functionality and expectations. Future efforts should concentrate on standardizing protocols for multimodal pain management, fostering shared decision-making between providers and patients, and implementing PPTFP. These interventions have the potential to enhance patient experience and recovery and mitigate the adverse effects of postpartum opioid use within the broader context of the opioid epidemic in the United States.

## Data Availability

N/A.

## Code Availability

N/A.
